# Association between Temperature Change and Outpatient Visits for Respiratory Tract Infections among Children in Guangzhou, China

**DOI:** 10.3390/ijerph120100439

**Published:** 2015-01-06

**Authors:** Yu Liu, Yong Guo, Changbing Wang, Weidong Li, Jinhua Lu, Songying Shen, Huimin Xia, Jianrong He, Xiu Qiu

**Affiliations:** 1 Division of Birth Cohort Study, Guangzhou Women and Children’s Medical Center, Sun Yat-sen University, 9 Jinsui Road, Guangzhou 510623, China; E-Mails: PH04LY@163.com (Y.L.); geyong084@163.com (Y.G.); liweidong30303@163.com (W.L.); dearlu@126.com (J.L.); shsy_22@163.com (S.S.); huiminxia@hotmail.com (H.X.); hjr0703@163.com (J.H.); 2 Department of Health Care, Guangzhou Women and Children’s Medical Center, Sun Yat-sen University, 9 Jinsui Road, Guangzhou 510623, China; 3 Central Laboratory, Guangzhou Women and Children’s Medical Center, Sun Yat-sen University, 9 Jinsui Road, Guangzhou 510623, China; E-Mail: changbingwang121@163.com

**Keywords:** temperature change, respiratory tract infections, outpatient visits, distributed lag non-linear model, children

## Abstract

The current study examined the association between temperature change and clinical visits for childhood respiratory tract infections (RTIs) in Guangzhou, China. Outpatient records of clinical visits for pediatric RTIs, which occurred from 1 January 2012 to 31 December 2013, were collected from Guangzhou Women and Children’s Hospital. Records for meteorological variables during the same period were obtained from the Guangzhou Meteorological Bureau. Temperature change was defined as the difference between the mean temperatures on two consecutive days. A distributed lag non-linear model (DLNM) was used to examine the impact of temperature change on pediatric outpatient visits for RTIs. A large temperature decrease was associated with a significant risk for an RTI, with the effect lasting for ~10 days. The maximum effect of a temperature drop (−8.8 °C) was reached at lag 2~3 days. Children aged 0–2 years, and especially those aged <1 year, were particularly vulnerable to the effects of temperature drop. An extreme temperature decrease affected the number of patient visits for both upper respiratory tract infections (URTIs) and lower respiratory tract infections (LRTIs). A temperature change between consecutive days, and particularly an extreme temperature decrease, was significantly associated with increased pediatric outpatient visits for RTIs in Guangzhou.

## 1. Introduction

Respiratory tract infections (RTIs) represent the most common type of illness in humans, and result in considerable morbidity, complications, and days lost from work and school [1]. It is estimated that in 2010, young children required hospitalization for 11.9 million episodes of severe and 3.0 million episodes of very severe acute lower respiratory infections, worldwide [2]. Additionally, RTI-associated pneumonia is the main cause of childhood mortality among children aged <5 years in developing countries [3,4].

In recent years, public health literature has increasingly recognized that ambient temperatures have a significant impact on the prevalence of pediatric respiratory diseases [5,6]. Compared to exposures to intermediate and comfortable temperatures, exposure to both extreme hot and cold weather is associated with increased morbidity from RTIs. Accordingly, current evidence suggests that the incidence of RTIs increases in temperate climates during the colder months of a year [7]. In one study, which included community-based data and hospitalization rates for children with respiratory diseases, both the community incidence and hospitalization rates for RTIs peaked during winter time and decreased during spring time [4]. In general, the most comfortable and safest temperatures exist in the range of 16.5 °C (representative of the Netherlands) to 29 °C (representative of Taiwan) [6,8].

Most previous studies have used a daily or monthly mean, maximum or minimum temperature as an indicator of exposure when evaluating the effects of temperature on RTIs. However, in contrast to the effects of absolute temperature, the effects of temperature variation have not been thoroughly investigated, and few studies have emphasized that large diurnal temperature changes may affect the respiratory system of humans, and especially children [9,10].

Although temperature may be associated with the incidence of RTIs, meteorological factors characteristic of different countries and geographic regions often vary, and may also be involved. Current knowledge about the effects of ambient temperature on RTIs mainly from developed countries [11]. Few studies have been carried out in developing countries in which population are more vulnerable to weather changes due to the lack of medical services. Guangzhou is one of the largest cities in southern China with a subtropical humid monsoon climate. RTIs (mainly pneumonia) are one of the top five causes of infant deaths and one of the main causes of admission to general pediatric wards in Guangzhou [12,13]. Nearly 90% of the pediatric patients with RTIs in Guangzhou were under five years old [14]. It was reported that the rate of pediatric admission due to upper respiratory tract infections (URTIs) was about 30% in Hong Kong [15]; a city that has a similar climate to that of Guangzhou. A better understanding of effects of climate change on respiratory health will provide useful information for public health. In this study, we hypothesized that a sharp temperature change between consecutive days in that location might be associated with an increased number of childhood RTIs. Therefore, data concerning outpatient visits were examined to determine whether a sharp temperature change might have been associated with the incidence and types of childhood RTIs reported in Guangzhou, China in the time period of 2012 to 2013.

## 2. Material and Methods

### 2.1. Study Setting

Guangzhou, located in South China, is the capital city of Guangdong Province and the third largest city in China. Guangzhou comprises 11 districts encompassing 7434 km^2^, and has 1.93 million under 18 years old according to the sixth national census conducted in 2010. It is situated at a latitude of 23°70′ N, and displays a typical subtropical climate with an average annual temperature of 22 °C.

### 2.2. Data Concerning Outpatient Visits for Children with Respiratory Tract Infections (RTIs)

All children who were aged <18 years during the time period studied, and had visited the Department of Pediatrics at Guangzhou Women and Children’s Medical Center for symptoms of an RTI were included in the study. Guangzhou Women and Children's Medical Center was formed by a merger between the original Guangzhou Children’s Hospital and the original Guangzhou Maternity and Child Care Hospital, and is the only first-class children’s hospital in the city of Guangzhou. Data on the daily number of outpatient visits for RTIs from 1 January 2012 through 31 December 2013 were collected and used for this study. An RTI was diagnosed based on guidelines in the Tenth Revision of the International Classification of Diseases (ICD-10) J00-J99, and the definition included URTIs, such as a common cold, otitis, pharyngitis, and sinusitis, as well as lower respiratory tract infections (LRTIs), such as bronchitis and pneumonia. RTIs diagnosed within two weeks of each other were considered as a single episode. 

### 2.3. Meteorological Data

Data concerning daily meteorological conditions in Guangzhou during the period of 1 January 2012 to 31 December 2013 were obtained from the Guangzhou Meteorological Bureau. Variables included the daily mean/maximum/minimum temperature (°C), relative humidity (%), and atmospheric pressure (hpa). Temperature change was defined as the difference in the mean temperature on two consecutive days [9,16].

### 2.4. Ethics Statement

This study was approved by the institutional ethical committee board of Guangzhou Women and Children’s Medical Center.

### 2.5. Statistical Analyses

Because outpatient and meteorological data were linked by date, there were lagging effects of temperature change. Thus a temperature on the current day, as well as changes on the preceding several days, could affect outpatient visits on the current day [17,18]. We utilized a distributed lag non-linear model (DLNM) to simultaneously explore non-linear and delayed relationships between the daily number of outpatient visits for RTIs ($Y_{t}$) and ambient temperature change [19,20,21]. The model was fitted using a generalized linear model with a quasi-Poisson family, which was specified as:

(1) $$\mathrm{Log}\left[E\left(Y_{t}\right)\right]=\alpha +\beta \cdot \text{Dow}+\gamma \cdot \text{Holiday}+\text{NS}\left(\text{Time},2\times 11\right)+\;\overset{20}{\underset{t=0}{\sum }}\text{NS}\left(\text{TC},4,\text{lag},4\right)+\text{NS}\left(\text{Tavg},\;3\right)+\text{NS}(\text{Hum},\;3)$$

Where E(Y*_t_*) is the expected number of outpatient visits for RTIs on day t (*t* = 1, 2, 3, …, 731) and α is the intercept. Day-of-the-week (Dow) and public holiday (PH) effects were initially considered for adjustment in this model, and the coefficients are denoted as β and γ, respectively. The natural cubic spline of time (Time) with 11 degrees of freedom (df) per year was used to control for long-time trends and seasonality. The mean temperature (Tavg) and relative humidity (RH) on any current day were corrected as baseline characteristics of ambient conditions, where 3 dfs were set [19]. When using this model, the effect of temperature change (TC) was examined through the choice of cross-basis along the two-dimensional space of temperature and lag days. Cubic splines were used in each dimension, and both dfs were set at 4. A 20-day maximum lag-time was established based on biological reasoning and a previously published empirical study that had included the incubation periods of common respiratory viruses [22]. The Akaike information criterion for quasi-Poisson models (QAIC) was used for determining the better-fit statistical model, and the model with the lower qAIC was selected.

We reported the relative risk (RR, with 95% confidence intervals (CIs)) created by extreme temperature changes (1% and 5% percentiles for temperature drop, and 95% and 99% percentiles for a temperature increase) for an outpatient visit for an RTI on specific lag days. A 0 °C temperature change was used as the reference value. Analyses stratified by age group and the type of respiratory disease were performed to identify subpopulations more susceptible to the effects of a large temperature change.

Risk estimates can be strongly influenced by the specifications included in the model used for a time series analysis; therefore, sensitivity analyses were conducted by changing dfs for the predictors. We also performed the analysis after changing the maximum lag-time to 14-day. Moreover, the exposure-response relationships were examined by the Poisson generalized additive model (GAM) [23] with the same predictors, in which dfs were determined by Akaike Information Criterion (AIC) [23,24], and the results were compared to those generated by DLNM.

All statistical tests were two-tailed, and *P*-values < 0.05 were considered statistically significant in terms of an exploratory data analysis. All statistical analyses were performed using the DLNM packages in R software Version 2.15.0 (R Development Core Team, 2012). 

## 3. Results

Statistical data regarding the daily number of outpatient visits for RTIs, daily temperature (including minimum, maximum, mean temperature, and temperature change), and relative humidity are summarized in Table 1. During the study period of 1 January 2012 to 31 December 2013, the average daily maximum, minimum, and mean temperatures were 26.3 °C, 18.6 °C, and 21.6 °C, respectively, while the mean temperature change was −0.01 °C. The average relative humidity in Guangzhou during the study period was 81.2%. There were totally 1,529,853 outpatient visits for RTIs, including 929,117 (60.7%) male and 600,590 (39.3%) female. The daily number of outpatient visits for RTIs ranged from 346 to 3486 (median, 2132), most of which were under six years old (85.3%) and diagnosed as URTIs (76.9%).

**Table 1:** Summary statistics of ambient temperature and outpatient visits for respiratory tract infections (RTIs) in Guangzhou.

Variables	Mean ± SD	Minimum	P25	Median	P75	Maximum
Maximum temperature (°C)	26.3 ± 6.5	7.2	21.9	27.7	31.5	36.8
Minimum temperature (°C)	18.6 ± 6.2	2.5	13	20	24.1	28.3
Mean temperature (°C)	21.6 ± 6.2	5.1	16.9	23	26.8	30.4
Temperature change (°C)^*^	−0.01 ± 1.93	−8.8	−0.9	0.2	1.1	5.8
Relative humidity (%)	81.2 ± 10.4	40	76	83	89	100
Daily number of hospital outpatient visits for respiratory diseases	2092.8 ± 479.6	346	1756	2132	2431	3486
**Group by Age (years)**						
0~	489.1 ± 124.0	103	401	481	572	1023
1~	664.6 ± 158.6	91	552	669	775	1116
3~	631.8 ± 172.7	87	516	634	755	1096
6~	307.4 ± 117.5	34	226	285	380	813
Group by Gender ^**^						
Male	1271.0 ± 285.8	222	1075	1287	1468	2120
Female	821.6 ± 196.4	124	688	835	956	1411
**Group by Diseases**						
Upper respiratory tract infection	1609.6 ± 375.5	252	1359	1620	1875	2558
Lower respiratory tract infection	483.3 ± 143.7	86	388	476	561	1077

^*^ The temperature change was defined as the difference of the current day's and the previous day’s mean temperature; ^** ^146 cases with missing gender.

**Figure 1:**
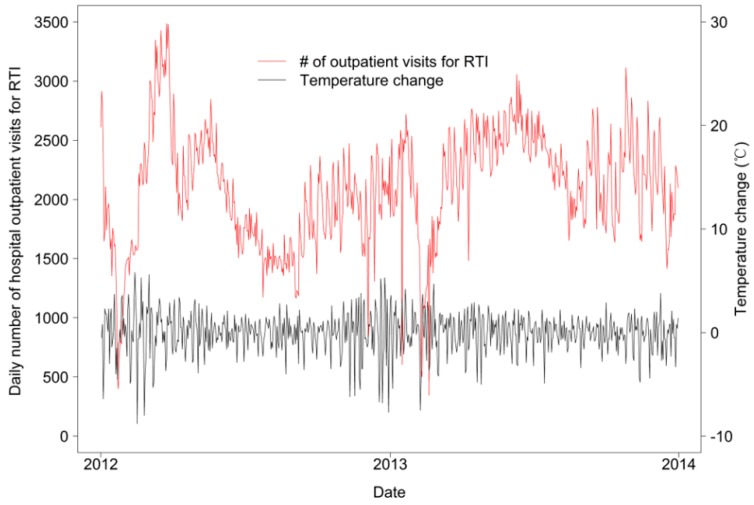
Daily number of outpatient visits for respiratory tract infections (RTIs) and temperature change versus time (January 2012–December 2013).

Trends in the daily number of outpatient visits for RTIs and temperature change during 2012–2013 are shown in Figure 1. The peak numbers of outpatient visits for RTIs occurred simultaneous with periods of relatively cold temperatures and large temperature fluctuations. The daily number of outpatient visits for RTIs appeared to decrease during periods of relatively stable temperatures.

**Figure 2:**
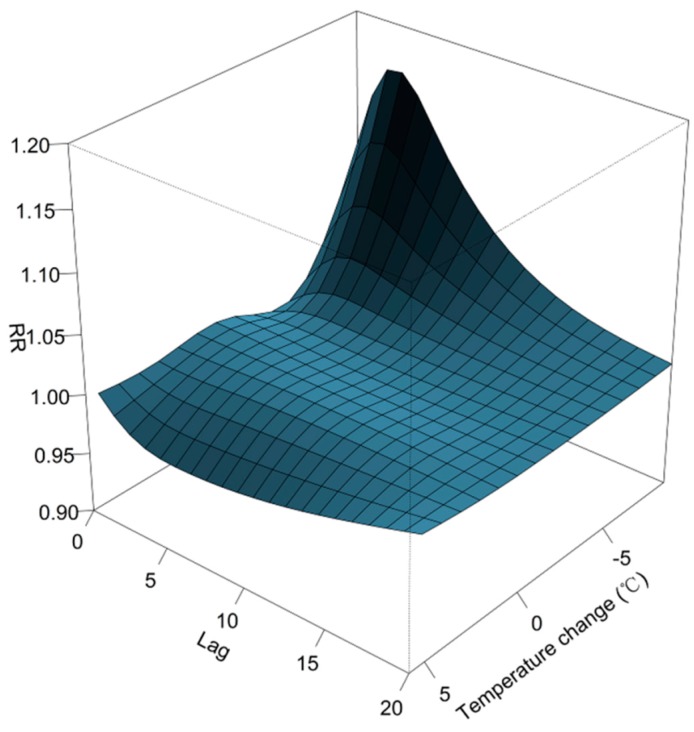
Relative risks of an outpatient visit for a respiratory tract infection (RTI) by temperature change (°C) and days of lag, with reference at 0 °C temperature change.

**Figure 3:**
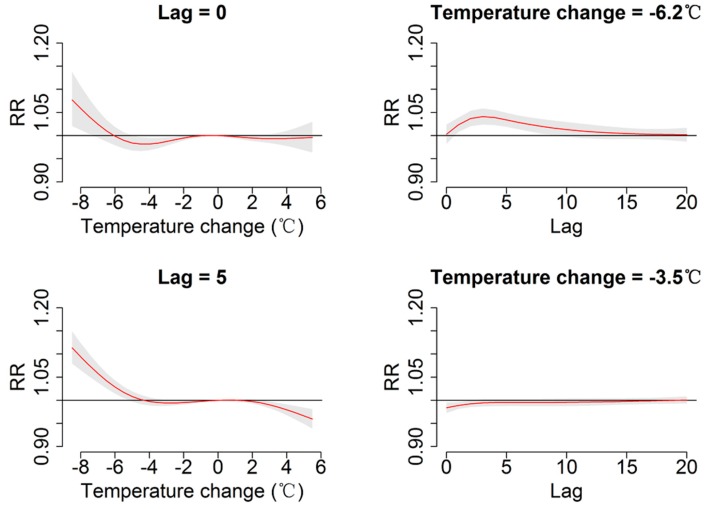

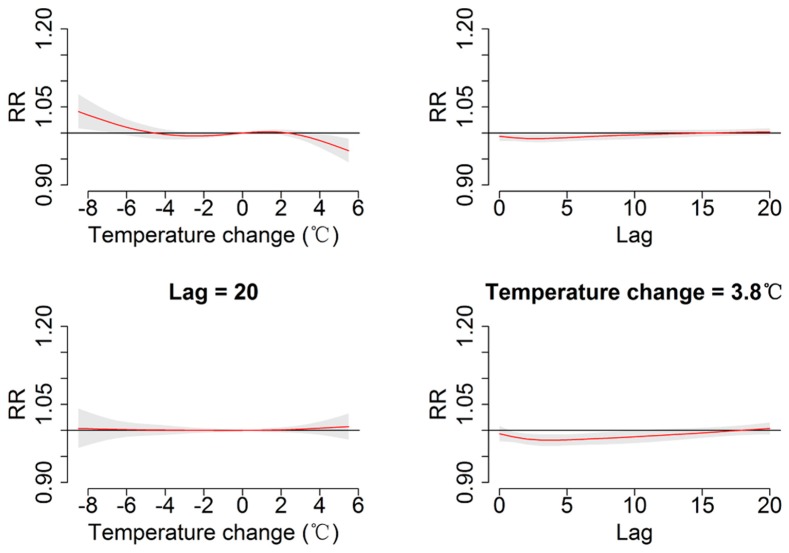
Plot of relative risk (RR) by temperature change at specific lags (**left**), RR by lag at 1st (−6.2 °C), 5th (−3.5 °C), 95th (2.9 °C) and 99th (3.8 °C) percentiles of temperature change distribution (**right**). The reference value was 0 °C temperature change.

**Figure 4:**
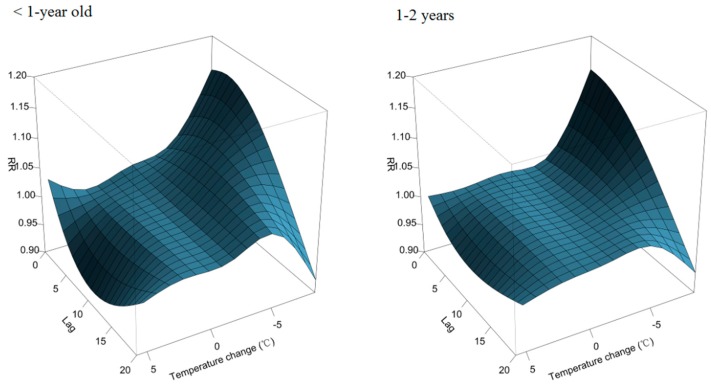

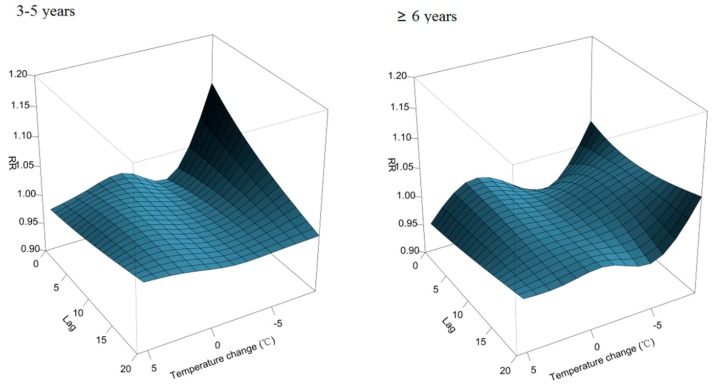
Relative risk of an outpatient visit for a respiratory tract infection (RTI) by temperature change (°C), days of lag, and subgroups of age. The reference value was 0 °C temperature change.

**Figure 5:**
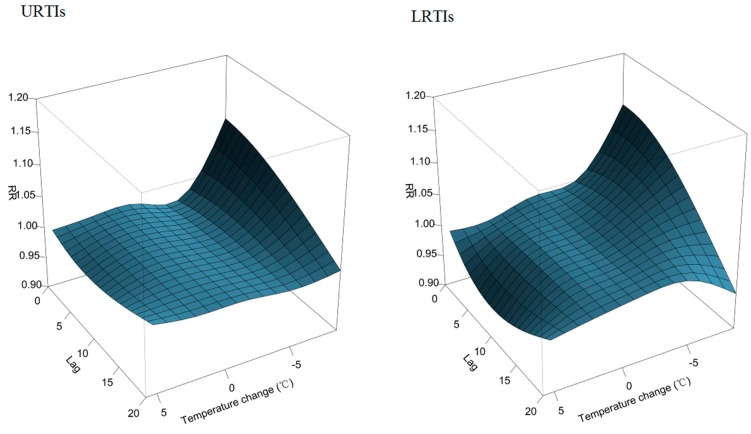
Relative risk of an outpatient visit for a respiratory tract infection (RTI) by temperature change (°C), days of lag, and RTI classification (upper RTIs (URTIs) and lower RTIs (LRTIs)). The reference value was 0 °C temperature change.

The overall effects of temperature change on daily outpatient visits for RTIs are depicted in Figure 2. The plot shows a nonlinear relationship between temperature and patient visits for RTIs. Inspection of the 3-D graph suggests that a large temperature decrease coincided with an immediate increase in the number patient visits for RTIs, while an increase coincided with a decreased number of visits. The maximum effect of a temperature drop (−8.8 °C) was reached at lag days 2~3 after its occurrence (RR: 1.16; 95% CI: 1.11, 1.22). As illustrated in Figure 3, the RR of RTIs by temperature change at specific lag periods (0, 5, 10, and 20 days) and by the lag at specific temperature changes (−6.2, −3.5, 2.9, and 3.8 °C), corresponded to approximately the 1st, 5th, 95th, and 99th percentiles, respectively, of the temperature change distribution in Guangzhou. The effects of temperature change on daily outpatient visits for RTIs changed with different lags periods, and the effect seemed to disappear at lag 20 (Figure 3, left). These results confirmed both the delayed effect of an extreme temperature decrease (−6.2 °C), and the significant resultant risk which lasted up to ~10 days (Figure 3, right). The association between temperature change and outpatient visits for RTIs by age group is presented in Figure 4. In Guangzhou, children aged ≤2 years, and especially those <1-year-old, were vulnerable to a sharp drop in temperature, while they were protected by a temperature increase. Children aged ≤2 years were most affected by a temperature change at lag days 10~15. The greatest effects of an extreme temperature decrease (−6.2 °C) on children aged <1-year and 1–2 years occurred on lag day five (RR: 1.08; 95% CI: 1.05, 1.11) and on the current day (lag day 0) (RR: 1.04, 95% CI: 1.01, 1.07), respectively (Table 2). A temperature change did not significantly affect the number of patient visits for RTIs by individuals aged >6 years. The relationship between temperature change and outpatient visits for URTIs and LRTIs at different lag periods is shown in Figure 5. While an extreme temperature decrease affected visits for both URTIs and LRTIs, the effects were more significant on outpatient visits for LRTIs.

In sensitivity analysis, we changed the dfs for time (6 to 10 per year), mean temperature (4 or 5) and relative humidity (4 or 5) and obtained similar results to those of original analysis. When we specified the maximum lag-time as 14-day, the shape of relationships between temperature and daily outpatient visits for RTIs did not change substantially, although the lag effect of the temperature decrease were prolonged (Figure S1). Finally, we compared the lag-specific risk estimates derived from GAM and DLNM models. Although the effect estimates obtained by GAM appeared to be attenuated, both methods provided similar patterns for the effect of temperature change (Figure S2).

**Table 2:** The effects of temperature change on different groups of outpatient visits for respiratory tract infections (RTIs), with 1%, 5%, 95% and 99% percentiles of temperature change relative to the reference 0 °C temperature change at different lag days in Guangzhou.

Lag (day)	RR (95% CI) for Temperature Change			
	−6.2 °C	−3.5 °C	2.9 °C	3.8 °C
**Overall**				
Lag0	1.00(0.98, 1.02)	0.98(0.97, 1.00)	0.99(0.98, 1.00)	0.99(0.98, 1.01)
Lag5	**1.03(1.02, 1.05)**	1.00(0.99, 1.00)	0.99(0.98, 1.00)	**0.98(0.97, 0.99)**
Lag10	**1.01(1.00, 1.03)**	1.00(0.99, 1.00)	1.00(0.99, 1.00)	0.99(0.98, 1.00)
Lag15	1.00(0.99, 1.02)	1.00(0.99, 1.00)	1.00(0.99, 1.01)	1.00(0.99, 1.01)
Lag20	1.00(0.99, 1.02)	1.00(0.99, 1.01)	1.00(1.00, 1.01)	1.00(0.99, 1.02)
**0 year**				
Lag0	**1.05(1.02, 1.09)**	**1.02(1.00, 1.03)**	**0.99(0.98, 1.01)**	**1.00(0.98, 1.03)**
Lag5	**1.08(1.05, 1.11)**	**1.03(1.01, 1.04)**	**0.98(0.96, 0.99)**	**0.96(0.94, 0.98)**
Lag10	**1.08(1.05, 1.10)**	**1.03(1.02, 1.05)**	**0.97(0.96, 0.99)**	**0.95(0.93, 0.97)**
Lag15	**1.05(1.03, 1.07)**	**1.03(1.02, 1.04)**	**0.98(0.97, 0.99)**	**0.97(0.95, 0.98)**
Lag20	1.01(0.98, 1.03)	**1.02(1.01, 1.04)**	1.00(0.99, 1.01)	0.99(0.98, 1.01)
**1–2 years**				
Lag0	**1.04(1.01, 1.07)**	1.00(0.98, 1.01)	0.99(0.98, 1.01)	0.99(0.98, 1.01)
Lag5	**1.04(1.02, 1.06)**	1.00(0.99, 1.01)	0.99(0.98, 1.00)	**0.98(0.96, 0.99)**
Lag10	**1.03(1.01, 1.06)**	1.00(0.99, 1.02)	0.99(0.98, 1.00)	**0.97(0.96, 0.99)**
Lag15	1.02(1.00, 1.04)	1.01(1.00, 1.02)	0.99(0.98, 1.00)	**0.98(0.97, 0.99)**
Lag20	0.99(0.97, 1.01)	1.01(1.00, 1.02)	0.99(0.98, 1.00)	0.99(0.97, 1.01)
**3–5 years**				
Lag0	0.99(0.96, 1.02)	**0.97(0.96, 0.99)**	0.98(0.97, 1.00)	0.98(0.96, 1.00)
Lag5	1.00(0.98, 1.02)	**0.98(0.97, 0.99)**	1.00(0.99, 1.01)	0.99(0.97, 1.01)
Lag10	1.00(0.98, 1.03)	0.98(0.97, 1.00)	1.01(0.99, 1.02)	1.00(0.98, 1.02)
Lag15	1.00(0.98, 1.02)	0.99(0.98, 1.00)	1.01(1.00, 1.02)	1.01(0.99, 1.02)
Lag20	1.00(0.97, 1.02)	0.99(0.98, 1.01)	1.01(1.00, 1.02)	1.01(1.00, 1.03)
**6–17 years**				
Lag0	0.97(0.92, 1.03)	**0.96(0.93, 0.98)**	1.00(0.97, 1.02)	0.98(0.94, 1.02)
Lag5	1.01(0.97, 1.06)	0.99(0.97, 1.02)	1.00(0.97, 1.02)	0.98(0.95, 1.02)
Lag10	1.02(0.98, 1.08)	1.01(0.98, 1.03)	1.00(0.97, 1.02)	0.99(0.95, 1.03)
Lag15	1.01(0.98, 1.05)	1.00(0.98, 1.02)	0.99(0.97, 1.01)	0.99(0.96, 1.02)
Lag20	0.99(0.95, 1.04)	0.98(0.96, 1.01)	0.99(0.97, 1.01)	0.99(0.96, 1.03)
**URTIs**				
Lag0	1.01(0.99, 1.03)	**0.98(0.97, 0.99)**	0.99(0.99, 1.00)	0.99(0.98, 1.01)
Lag5	1.01(1.00, 1.03)	0.99(0.98, 1.00)	0.99(0.99, 1.00)	0.99(0.98, 1.00)
Lag10	1.01(1.00, 1.03)	0.99(0.98, 1.00)	1.00(0.99, 1.00)	0.99(0.98, 1.00)
Lag15	1.00(0.99, 1.02)	0.99(0.99, 1.00)	1.00(0.99, 1.00)	1.00(0.99, 1.01)
Lag20	0.99(0.98, 1.01)	0.99(0.99, 1.00)	1.00(0.99, 1.01)	1.00(0.99, 1.02)
**LRTIs**				
Lag0	**1.04(1.01, 1.07)**	1.01(0.99, 1.02)	0.98(0.97, 1.00)	0.98(0.97, 1.00)
Lag5	**1.05(1.03, 1.07)**	**1.01(1.00, 1.03)**	**0.98(0.97, 0.99)**	**0.97(0.95, 0.98)**
Lag10	**1.04(1.02, 1.07)**	**1.01(1.00, 1.03)**	**0.98(0.97, 0.99)**	**0.96(0.95, 0.98)**
Lag15	**1.02(1.00, 1.04)**	**1.01(1.00, 1.02)**	0.99(0.98, 1.00)	**0.97(0.96, 0.99)**
Lag20	0.99(0.97, 1.01)	1.01(1.00, 1.02)	0.99(0.98, 1.00)	0.99(0.97, 1.00)

## 4. Discussion

In the present study, a novel DLNM was used to examine the association of daily outpatient visits for childhood RTIs with temperature changes in Guangzhou. The DLNM allows for estimation and statistical testing of a non-linear and delayed effect [25,26]. Our results showed the association between RTIs and temperature was non-linear. We demonstrated that a relatively large decrease in temperature between consecutive days increased the risk of outpatient visits for RTIs. Lagged effects of temperature change on outpatient visits among children with RTIs were also observed. The delayed effect of a temperature decrease could last ~10 days, and the maximum effect of a temperature drop was seen on lag days 2~3. Children aged 0–2 years, and especially those aged <1 year, were particularly vulnerable to the effects of a temperature drop on their respiratory health. 

In our study, the harmful health effects of a temperature decrease were generally in accordance with those reported in previous studies, which showed that a temperature decrease conferred an increased risk for RTIs. Most available evidence suggests that exposure to cold is associated with an increased incidence of RTIs, and this finding has important public health implications [1,27,28,29]. A previous retrospective study of medical records showed that several meteorological parameters (including low temperatures) were associated with an increased occurrence of acute laryngitis [30]. Hashimoto *et al*. reported a positive association between a rapid decrease of temperature within a three-day period, and an increase in pediatric asthma emergency visits in Tokyo [31]. Another study conducted in Japan found a positive association between within day temperature change and asthma among children aged <12 years [32]. Finally, Xu *et al*. reported that a sharp temperature decrease between consecutive days had an adverse impact on cases of childhood pneumonia in Brisbane [33]. Although changes in temperature have been shown to adversely affect childhood respiratory-related health, a great heterogeneity (vulnerable population, confounding factors, lag time) exists among such studies due to their varied designs and statistical methods [34]. For instance, lag days in prior studies ranged from the day of exposure to an event (lag 0) to 27 days after exposure [21,35,36]. We found that children aged 0–2 years were vulnerable to a drop in temperature, and the effect of the temperature decrease could be manifested up to ~10 days later. However, Kan *et al*. found no adverse effects of a temperature change on children aged 0–4 years [37]. Such a difference in effects might be partially explained by the different biological processes and mechanisms induced by a temperature change [38].

The mechanisms underlying the effects of temperature change on RTIs have not been fully understood. Most of the available evidence from laboratory and clinical studies suggested that temperature can directly influence RTIs in children by affecting inflammation pathways or pathophysiological responses, such as vasoconstriction in the respiratory tract mucosa and suppression of immune responses [38,39,40,41,42]. A sudden temperature change might lead to pathophysiological responses of the respiratory epithelium, such as bronchospasms and inflammatory changes, which could trigger an RTI [38]. Exposure to cold air could induce an increased number of granulocytes and macrophages in the lower airways, and was possibly associated with the development of RTIs [41]. The effect of cooling of the nasal airway on the susceptibility to RTIs was also reported [43,44,45]. Koskela *et al.* showed that the cooling of the skin of the face might be the trigger for the bronchoconstriction during resting nasal ventilation at cold air exposure [44]. In addition, temperature may also indirectly affect RTI triggers, such as viral infections, bacterial activity, and time spent outdoors, hence the risk of RTIs [46,47]. The vulnerability to temperature variations displayed by children might partially be attributed to several different factors. First, their relatively less-developed thermoregulation capability and greater metabolic rate may render children more sensitive to extreme temperature change [48,49]. Furthermore, most children, and especially those aged <1-year, cannot care for themselves, and are dependent on others to protect them from environmental change [5]. Basu *et al.* found that infants were more likely to be impacted by temperature change due to their inappropriate thermoregulatory response [50]. In the current study, we also found that infants (aged <1-year) were more sensitive to a temperature decrease compared to other age groups. Meanwhile, our results suggest that in children, LRTIs are more sensitive to temperature change compared to URTIs, partially due to differences in viral activity. 

Certain limitations of this study should be acknowledged. First, this was an ecological study, so individual exposure data were not available, and some degree of measurement bias was inevitable. Second, air pollution, which could also accelerate the development of RTIs and is associated with ambient temperature [51,52,53], was not adjusted in the models because of lack of data. Thus, the effect estimates for association of temperature with RTIs might be biased. Third, the time series in present study were short (two years). We did not collect data on longer time series and unable to control the influence of exceptional seasons on the effect estimates. However, our results indicate that temperature change was associated with an increased number of outpatient visits for RTIs in children, and this information is important from a public health perspective and for planning appropriate risk management strategies. Additionally, our results were adjusted for the mean temperature and season on a current day, and suggest that temperature changes between adjacent days might be used as a predictor of outpatient visits for RTIs.

## 5. Conclusions

In summary, we found a significant relationship between temperature change and pediatric outpatient visits for RTIs in Guangzhou, China. A sharp temperature decrease between consecutive days was associated with an increased risk of pediatric outpatient visits for RTIs. Additionally, the association between a temperature change and the risk for a clinical visit appeared to differ by patient age and RTI classification. Our findings suggest that risk management strategies for maintenance of public health should focus on the effects of temperature change. 

## Supplementary Files

Supplementary File 1
