# Supplementary material for: Association between Temperature Change and Outpatient Visits for Respiratory Tract Infections among Children in Guangzhou, China
Source: Int J Environ Res Public Health. 2015 Jan 6;12(1):439–54. doi: 10.3390/ijerph120100439 (PMC4306872; doi:10.3390/ijerph120100439)
Supplement: Supplementary File 1 [file ijerph-12-00439-s001.pdf]

Supplementary Information

Association between Temperature Change and Outpatient Visits for Respiratory Tract Infections among Children in Guangzhou, China

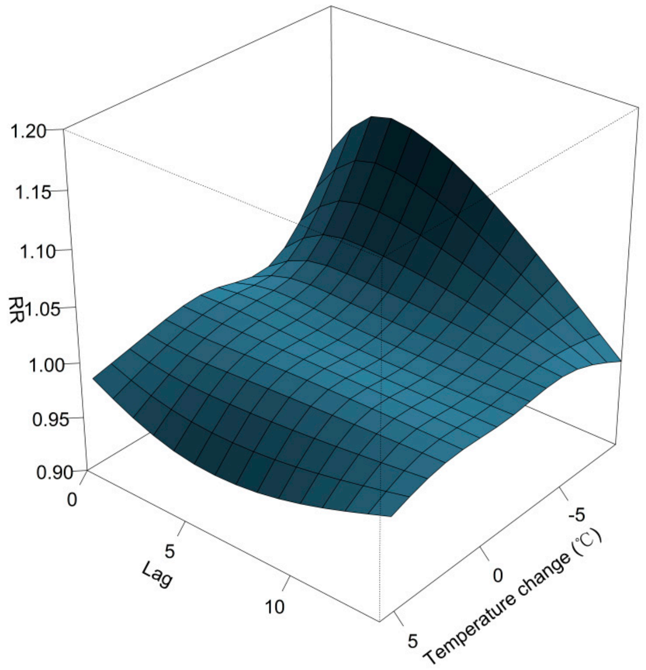

Figure S1. 3-D plot of RRs along temperature change (°C) and lags, with reference at 0 °C and a 14-day maximum lag time.

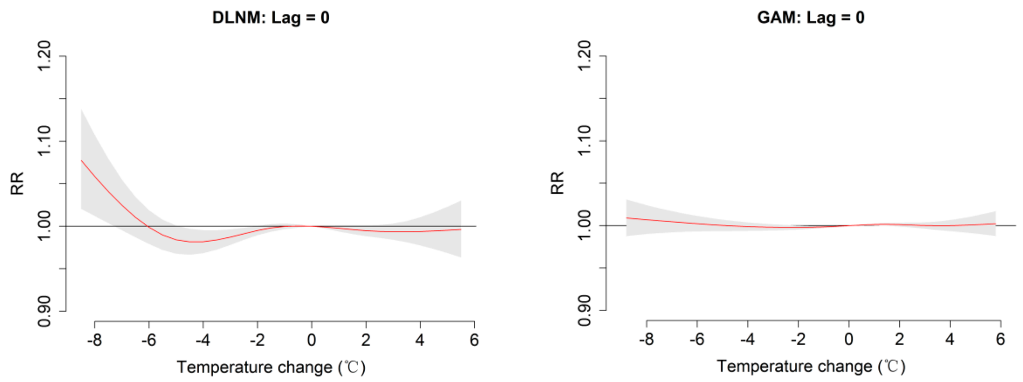

Figure S2. *Cont.*

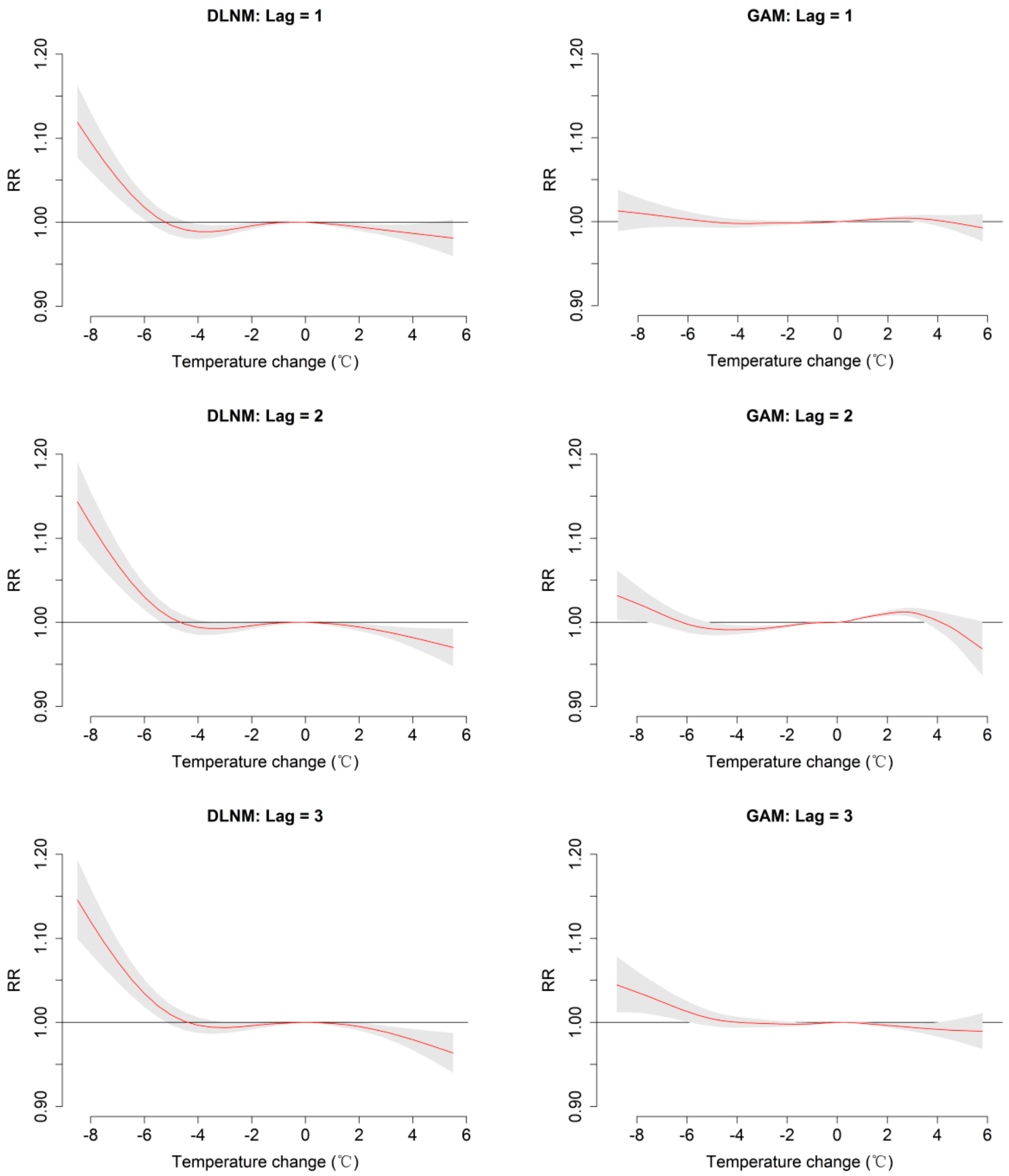

Figure S2. Comparison of lag-specific RRs generated by DLNM models and GAM models.

© 2015 by the authors; licensee MDPI, Basel, Switzerland. This article is an open access article distributed under the terms and conditions of the Creative Commons Attribution license (http://creativecommons.org/licenses/by/4.0/).
